# Progressive Slurred Speech as an Atypical Presentation of GDAP2-Related Spinocerebellar Ataxia: A Case Report

**DOI:** 10.7759/cureus.113246

**Published:** 2026-07-23

**Authors:** Norah Aljalal

**Affiliations:** 1 Neurology, Imam Abdulrahman Al Faisal Hospital of National Guard, Dahran, SAU

**Keywords:** exome sequencing, gdap2, hereditary ataxia, progressive dysarthria, scar27, spinocerebellar ataxia

## Abstract

A 47-year-old man with diabetes mellitus and dyslipidemia developed progressive slurred speech over four years. MRI revealed no cerebellar atrophy, and routine laboratory and neurophysiological investigations were unremarkable. His brother exhibited similar but advanced symptoms, including dysphagia and severe gait impairment, leading to wheelchair dependence. Whole-exome sequencing identified a likely pathogenic homozygous nonsense mutation in the GDAP2 gene, confirming a diagnosis of autosomal recessive spinocerebellar ataxia type 27 (SCAR27). This report emphasizes the importance of considering hereditary ataxias in the differential diagnosis of progressive dysarthria, even in the absence of striking ataxic signs or neuroimaging abnormalities. Early genetic testing can prevent unnecessary investigations, facilitate appropriate counselling, and improve the understanding of rare neurological conditions. Identifying novel mutations contributes to delineating the full clinical spectrum of SCAR27 and may guide research into targeted therapeutic strategies.

## Introduction

Dysarthria, also known as slurred speech, is a common neurological symptom that can lead to referral to a specialist. It results from the loss of motor control over muscles involved in speech, which include impairments in articulation, phonation, resonance, and prosody [1]. Although nonspecific, it is a significant finding in clinical practice because it may be the first sign of a wide range of disorders. These include both acute acquired conditions, such as cerebrovascular accidents, and slowly progressive neurodegenerative or inherited disorders [2]. Diagnosing the various underlying causes is especially challenging when dysarthria is the initial or main symptom.

Among the most commonly employed causes of progressive dysarthria in middle-aged adults are acquired lesions (especially small-vessel disease or infarction of the brainstem) followed by demyelinating lesions such as multiple sclerosis, motor neuron disease (amyotrophic lateral sclerosis), and neurodegenerative lesions such as Parkinson's disease and atypical Parkinsonian syndromes [3]. Myasthenia gravis (neuromuscular junction disorder) can also be associated with dysarthria, which has diurnal variation. Additional considerations are metabolic causes, which include vitamin E deficiency, Wilson's disease, or thyroid dysfunction [4].

However, if the preliminary examination does not reveal the underlying cause, it should be considered to be hereditary etiologies, such as genetic disorders, especially Friedrich ataxia or spinocerebellar ataxias (SCAs). Unlike acute cerebrovascular or inflammatory causes, hereditary disorders tend to have a gradual, insidious course, often in the context of a positive family history.

Spinocerebellar ataxias: clinical and genetic overview

Spinocerebellar ataxias (SCA) are a heterogeneous group of neurodegenerative diseases that mainly manifest as progressive gait instability, clumsiness of limbs, and speech impairment [5]. Dysarthria in SCAs typically occurs in conjunction with other cerebellar symptoms, including gait instability or disturbances in oculomotor function [5]. Nevertheless, the presence of phenotypic variability is a characteristic of the disease category, and manifestations in which dysarthria present earlier have been reported in a recent study [6].

However, over 40 genetic variations of SCAs have been characterized to date, with a variety of pathogenic mechanisms that include repeat amplifications, missense mutations, and truncating mutations of neuronal survival, mitochondrial, and synaptic signaling genes [7]. Traditionally, trinucleotide repeat expansion in the ATXN1, ATXN2, and ATXN3 family of genes was thought to be the most common etiological factor. Nevertheless, following the development of next-generation sequencing, a host of new genes have been linked to it, including GDAP2 [8].

GDAP2-related ataxia

The GDAP2 protein is involved in cellular stress response pathways, and loss-of-function mutations have been associated with increased neuronal vulnerability and cerebellar degeneration [9]. The mutations in GDAP2 have been associated with a rare autosomal recessive form of ataxia termed spinocerebellar ataxia type 27 (SCAR 27). Symptoms reported included dysarthria, ataxia, stiffness, cognitive decline, and cerebellar atrophy as observed on imaging [10]. Moreover, solitary or excessively severe dysarthria is rare, but a combination of gait ataxia and speech impairment is prevalent. Recognition of such rare conditions is important, since they may be mistaken for non-hereditary conditions. Besides, SCAR 27 being rare, any new case contributes to increasing our understanding of the range of its phenotypes [11].

Diagnostic challenges and the importance of genetic testing

Identification of hereditary ataxias, especially rare ataxia forms, is still a neuronal problem. This is, in part, due to their low prevalence rate and also due to their symptom similarity to other more prevalent disorders. Without incredible cerebellar symptoms or imaging presentation, physicians might not pay attention to genetic etiology as early as they need to and therefore delayed diagnosis occurs. Traditional diagnostic means, such as MRI and neurophysiological ones, tend to be usual at an initial stage of the disease [12]. Whole-exome sequencing has significantly improved diagnostic yield in unexplained neurological disorders [13].

Diagnosis has been transformed by the introduction of next-generation sequencing, particularly whole-exome sequencing (WES). Thousands of genes can be simultaneously interrogated using WES, which can improve the diagnostic outcome in patients with idiopathic neurological syndromes [13]. In cases that could not be well established clearly by the normal investigations, which was the case, WES provided the definitive diagnosis. Importantly, genetic diagnosis not only sheds light on the definition of the clinical manifestation but also enables the offer of the concept of genetic counseling to such families, apart from informing them of the opportunity of introducing further specific gene-targeted therapies [14].

Rationale for the present case report

The case under consideration is of clinical significance for a number of reasons. First, it demonstrates a rare expression of GDAP2-related ataxia where the main and primary symptoms were progressive dysarthria, and gait ataxia was mild and spared invalidity over several years. Second, the absence of cerebellar atrophy and hence lack of MRI is that neuroimaging can also be healthy in early or unconventional instances of SCAR27. Third, the positive family history demonstrates the essence of a comprehensive evaluation of the pedigree to indicate the direction of the diagnostic suspicion. Lastly, a new GDAP2 nonsense mutation was discovered, which contributes to the increasing range of pathogenic variants and enriches the spectrum of previously known phenotypes of SCAR27.

Hereditary ataxias, in the phenomenon of progressive speech disorders, must be mentioned in the list of differentials, according to the characteristics of this illness in middle age, regardless of the lack of the usual features, such as cerebellar atrophy or the most severe complication in gait disorders. It also notes the importance of genetic testing with regard to the description of rare neurological pathological processes. By documenting and analyzing such bizarre manifestations, clinicians and researchers will have a more accurate view of the dynamic SCAR27, improved accuracy in the diagnosis, and, ultimately, a more productive impact on patient care.

## Case presentation

An older man aged 47 years, serving as a soldier and having a background history of type 2 diabetes mellitus and dyslipidemia, presented with a complaint of poor, slurred speech that is progressing over four years. His diabetes and dyslipidemia were well controlled using oral hypoglycemic and lipid-lowering agents. No history of a previous cerebral event or traumatic brain injury, as well as no exposure to neurotoxic substances, was identified.

History of present illness

The first symptom was a mild change in the quality of speech. Family members noticed a change in the patient's articulation and the consonant pronunciation became more problematic. The speech was reported to be monotonous, slow, and increasingly difficult to comprehend. 

Although the speech disturbance was progressive, no dysphagia, aspiration, or nasal regurgitation was noted, A mild cough in the process of swallowing thin liquids was reported three years later, but in general, swallowing was still intact. No visual symptoms, including diplopia and blurred vision, occurred. The patient did not complain of weight loss, limb weakness, muscle wasting, fasciculation, sensory symptoms, or incontinence of bladder and bowel. There were no reported variations in predominantly diurnal symptoms, which is why neuromuscular junction disorders were less probable.

The gait was maintained till the early stage, but slight instability was also observed later, especially when walking on uneven surfaces. Independent ambulation and work-related activities were acceptable. There was no history of cognitive impairment.

Family history

The family history revealed that there was a younger brother, age 42 years old, who presented with similar symptoms, but with earlier onset of ataxia and dysarthria at the age of 36. The consequence of these problems includes the development of dysphagia with progressive gait ataxia, resulting in his dependence on a wheelchair by the age of 40. The parents did not show any similar symptoms that would move towards an autosomal recessive inheritance pattern. Family history did not show any known consanguinity-related members of the family, as there had been no other family members diagnosed with hereditary ataxias or neurodegenerative disorders.

**Figure 1:**
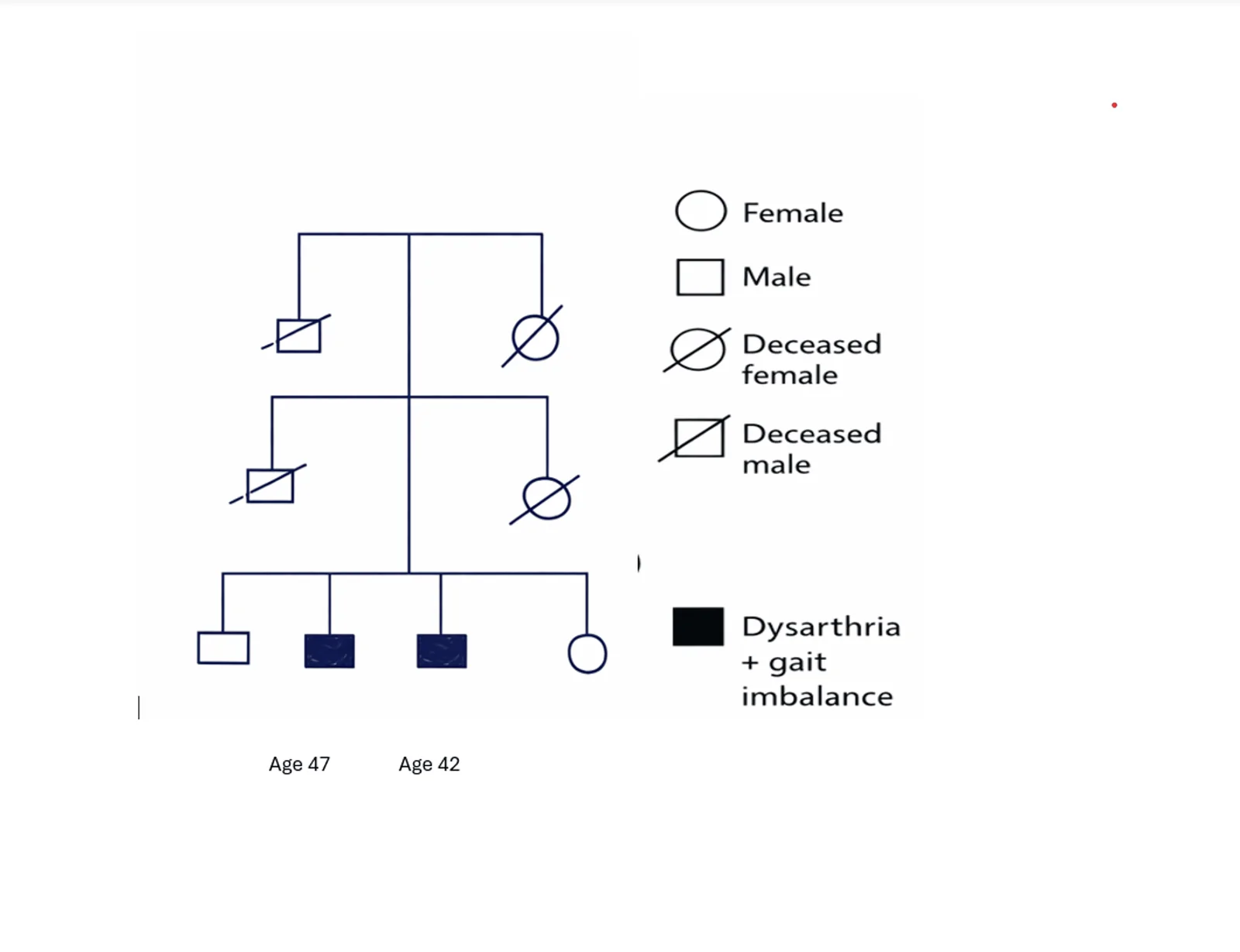
Pedigree diagram of family history. Two affected brothers with dysarthria and gait imbalance.

Neurological examination

Upon examination, the patient was alert and oriented to time, place, and person with normal higher mental abilities. There was a notable speech deficiency, slow, monotonic production with low articulation, which is indicative of cerebellar dysarthria. Neurological examination of cranial nerves demonstrated normal movements of the eyes with no nystagmus or ptosis. The tongue was midline, without fasciculations or atrophic appearance, with the palate raised equally. There was no bulbar palsy.

Motor exam showed the presence of normal bulk and tone of all four extremities, with full strength being observed 5/5 on the Medical Research Council (MRC) scale. None of the involuntary movements, tremors, or dystonic postures. Reflexes in lower and upper limbs were brisk (grade 3+) without progressive occurrence of clonus. His plantar responses were bilateral flexor. Also, there was preservation of sensory features of touch, pain, vibration, and proprioception.

The cerebellar examination indicated that the patient has poor performance on tandem gait and heel-to-shin tests, on the left side. Finger-to-nose test was normal. Romberg's test was negative. The patient could walk without walking aids.

Systemic examination

No significant changes were noted in abdominal, respiratory, and cardiovascular examination. No skin, skeletal, or scoliosis or winging of the scapula was found. The muscles were not atrophied and had no fasciculations. The risk of an inherited disorder increased due to a strong family history (particularly a sibling who was affected and showed more severe symptoms). The reliance on the lack of motor neuron manifestation, the presence of sensory impairment, diurnal variations, or metabolic anomalies all pointed to the rejection of more prevalent acquired factors.

Treatment and follow-up

The patient was managed conservatively with supportive care. Speech therapy was initiated to improve communication, but he stopped after a few sessions as he didn't feel much improvement. He was also referred to physiotherapy to improve his gait, but he did not follow up with the physiotherapist. According to the patient, he is satisfied with his gait and motor power. His diabetes and dyslipidemia were controlled with oral medications: metformin 500 mg twice daily and atorvastatin 20 mg once daily. On follow-up, the patient showed slow progression of dysarthria with mild gait instability but remained independently ambulatory with no dysphagia. No new neurological deficits were observed.

Investigations

To exclude acquired, metabolic, and structural causes, a thorough panel of investigations was carried out to assess progressive dysarthria and moderate cerebellar signs before considering genetic testing.

Neuroimaging

**Figure 2:**
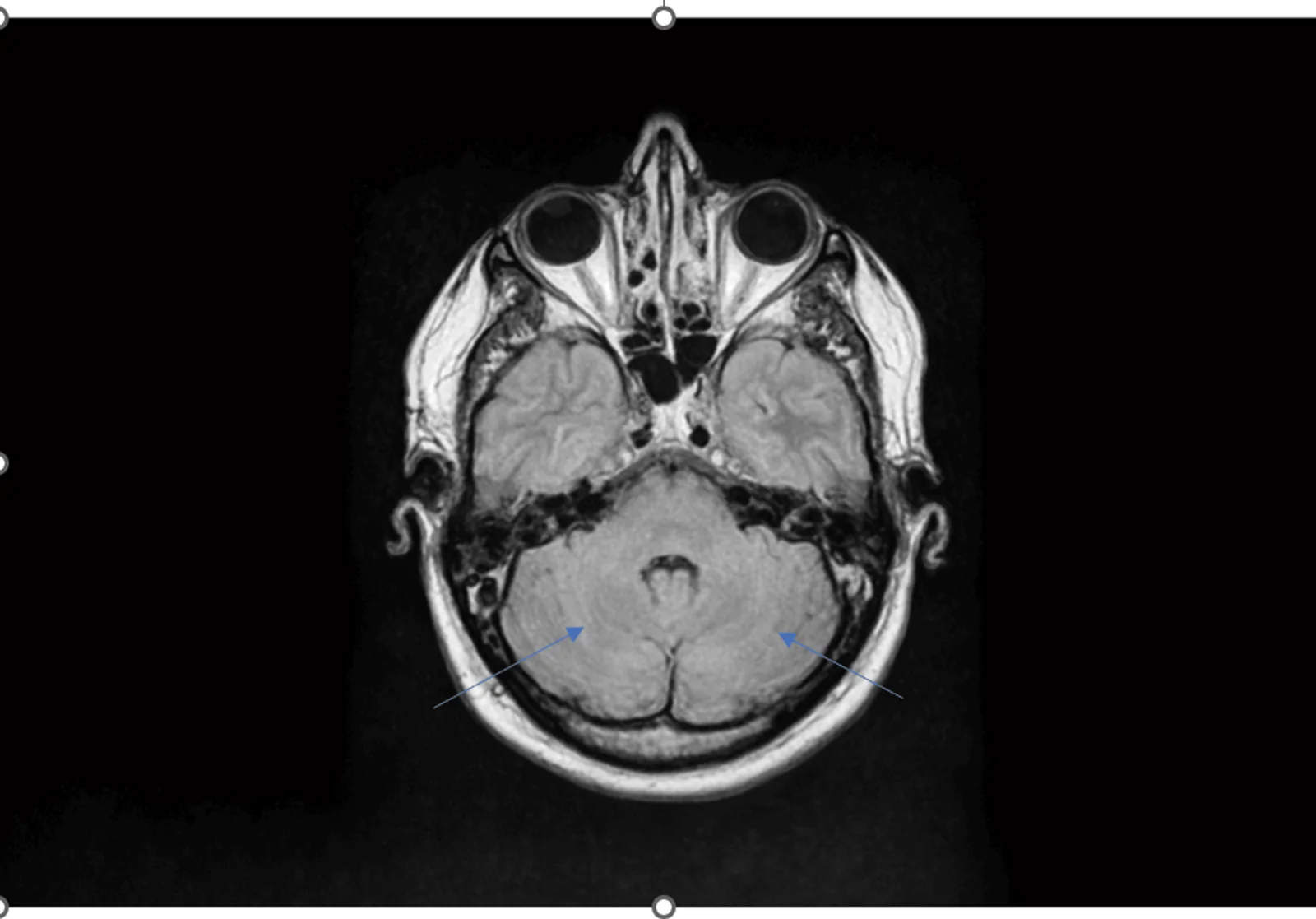
T1 brain MRI (magna (axial section)). Blue arrows identify the bilateral cerebellar hemispheres The brain MRI shows normal brain structure of bilateral cerebellar hemispheres with no lesions or atrophy identified.

**Figure 3:**
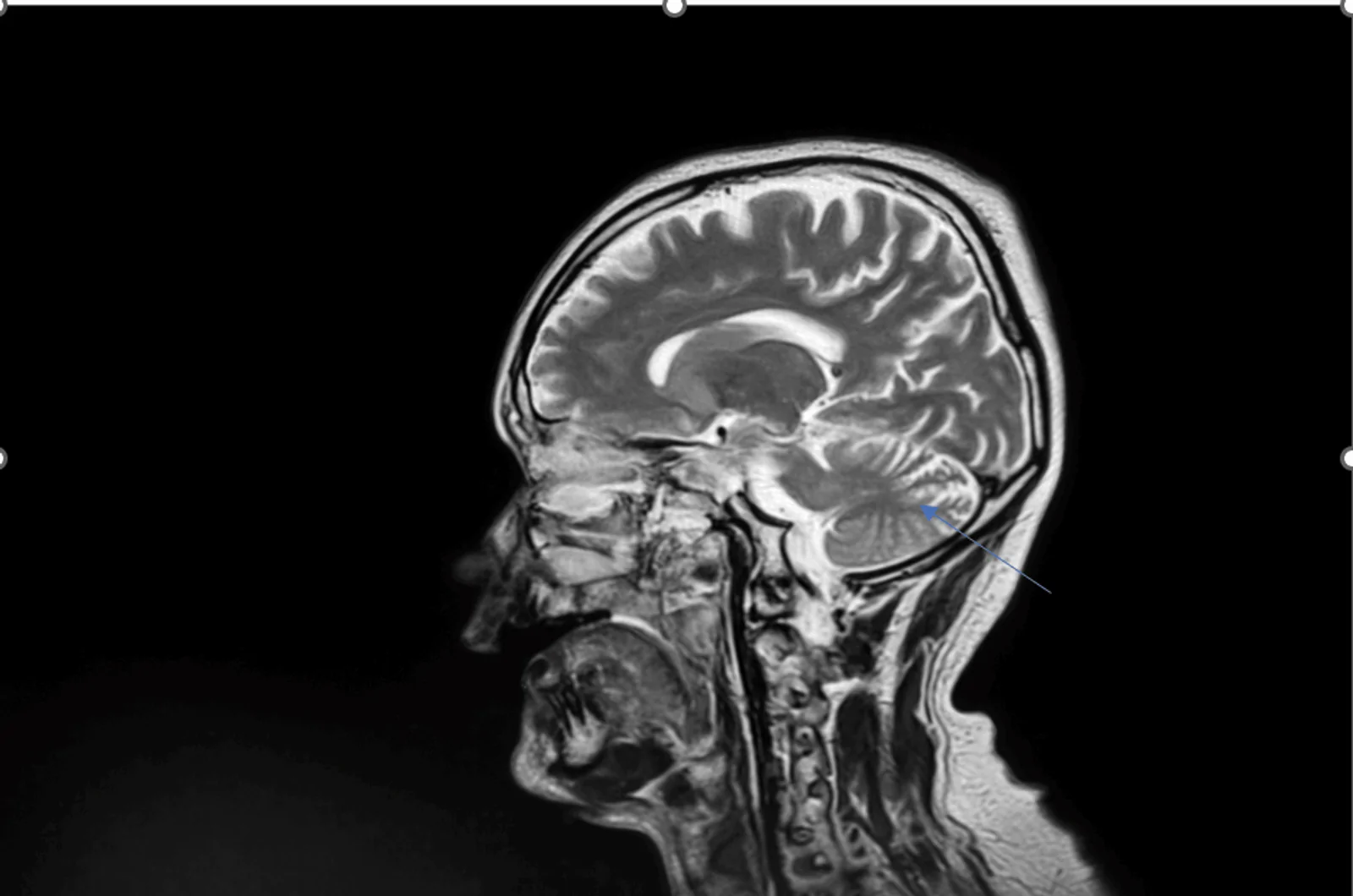
Brain T2 sagittal section, with arrow identifying the cerebellum with normal signal intensity.

Brain magnetic resonance imaging (MRI), including T1-weighted axial and T2-weighted sagittal sequences, revealed no signal abnormalities (Figures 2, 3). No instances of cerebellar hemisphere, vermis, or cerebral white matter signal abnormalities were observed. No subcortical or cortical ischemic change, demyelination, or space-occupying lesions and no cerebellar or brainstem atrophy was noted. Usually, cerebellar atrophy is observed in hereditary ataxias, which exhibit a volume-depleting effect on the progression of the disorder. This common imaging finding further complicated the diagnostic process, as it could be misinterpreted to suggest a non-degenerative etiology of the disease at its early onset [15]. Although this patient had his symptoms for four years, no clear atrophic changes of the cerebellum were observed.

Nerve conduction study

Nerve conduction studies (NCS) of lower-limb parameters were within normal limits, with no evidence of demyelination or axonal loss. The detailed NCS findings are summarized in Tables 1, 2.

**Table 1:** Lower limb NCS (motor tibial and fibular nerves bilateral) Reference ranges (tibial and fibular nerves): Distal latencies <6.5 ms, CMAP amplitude >2 mV, conduction velocity >40 m/s (lower limb); F-wave latency <31 C (tibial/fibular). All values within normal limits with no demyelination or axonal damage to peripheral nerves. CMAP: compound muscle action potential.

Nerve	Side	Distal Latency (ms)	CMAP Amplitude (mV)	Conduction Velocity (m/s)	F-Wave Latency (ms)
Tibial nerve (motor)	Right	4.1	10.2	48	48.3
Tibial nerve (motor)	Left	4.2	9.8	47	49.1
Common fibular nerve (motor)	Right	3.9	4.8	46	47.6
Common fibular nerve (motor)	Left	4.0	4.6	47	48.2

**Table 2:** Sensory nerve conduction study of bilateral sural nerve. Recording site: posterior ankle, stimulation site: posterior-lateral calf. Reference ranges of sural nerve conduction study normal values: Peak latency <4 ms, amplitude >5 μV, conduction velocity >35 m/s. All values within normal limits. Abbreviations: CV: conduction velocity; Norm: Normal value; lateral mall: lateral malleolus.

Nerve	Site	Latency (peak) (ms)	Norm	Amplitude (p‑p) (µV)	Norm	Segment	Distance (cm)	CV (peak) (m/s)	Norm
Left Sural Sensory	Calf - lateral malleolus	2.4	< 4.0	20	> 5.0	Calf - lateral mall	14	58	> 35
Right Sural Sensory	Calf - lateral malleolus	2	< 4.0	28	> 5.0	Calf - lateral mall	14	70	> 35

**Figure 4:**
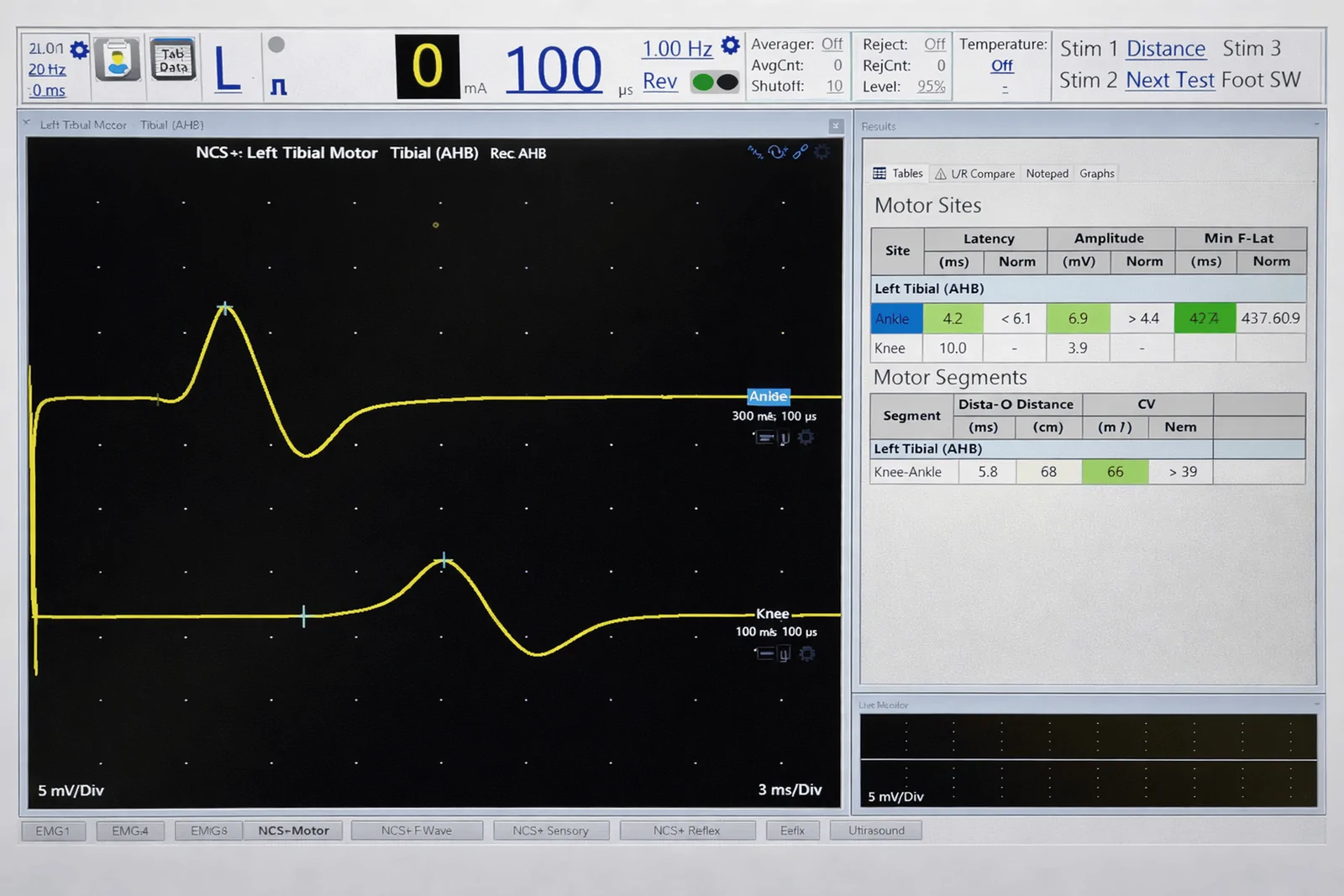
Left tibial motor nerve conduction study recording from abductor hallucis brevis (AHB) muscle: stimulation at ankle, popliteal fossa

**Figure 5:**
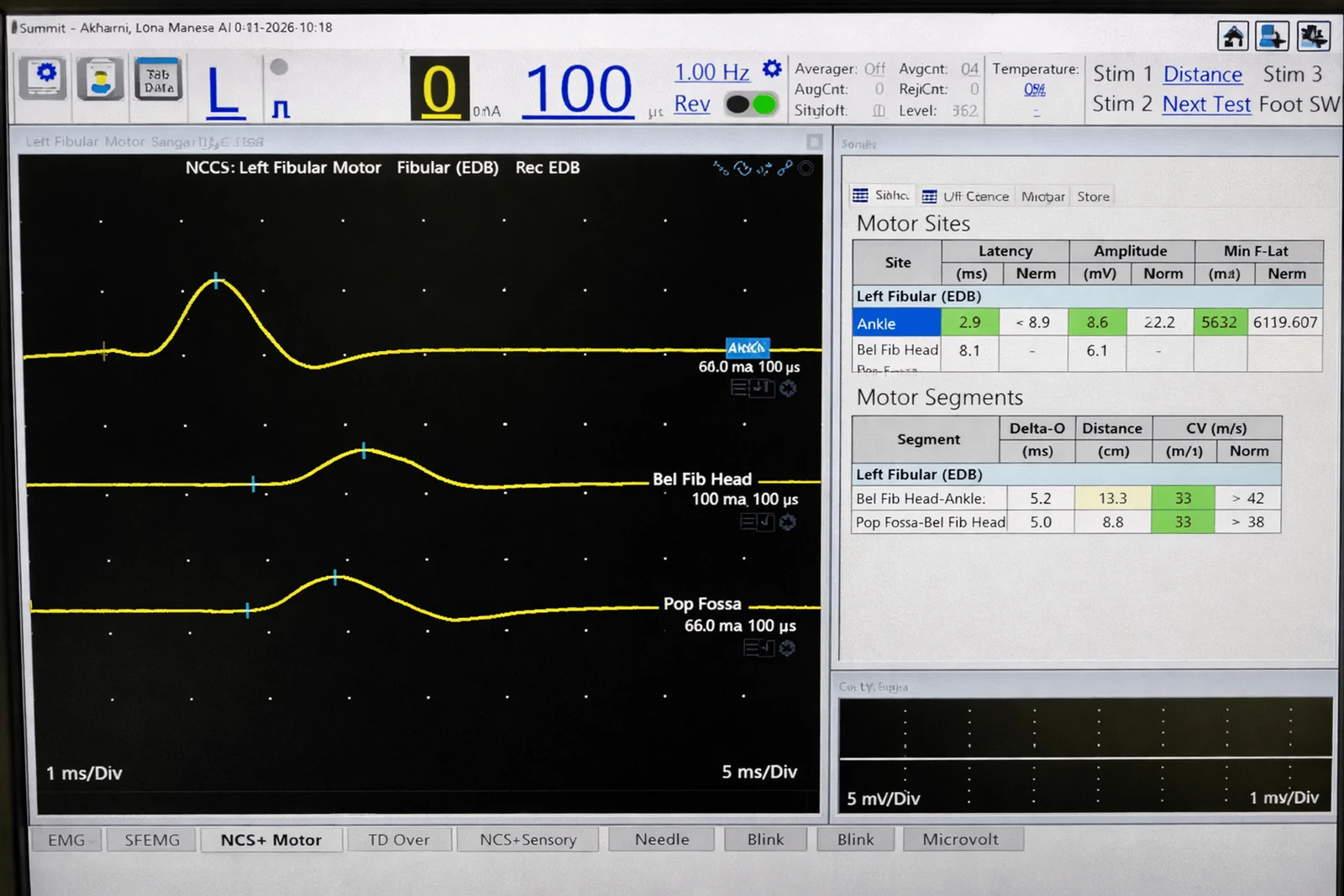
Left fibular motor nerve NCS recording from (EDB) muscle. Stimulation sites: ankle, below fibular head, and above fibular neck NCS: Nerve conduction study; EDB: extensor digitorum brevis (EDB) muscle.

**Figure 6:**
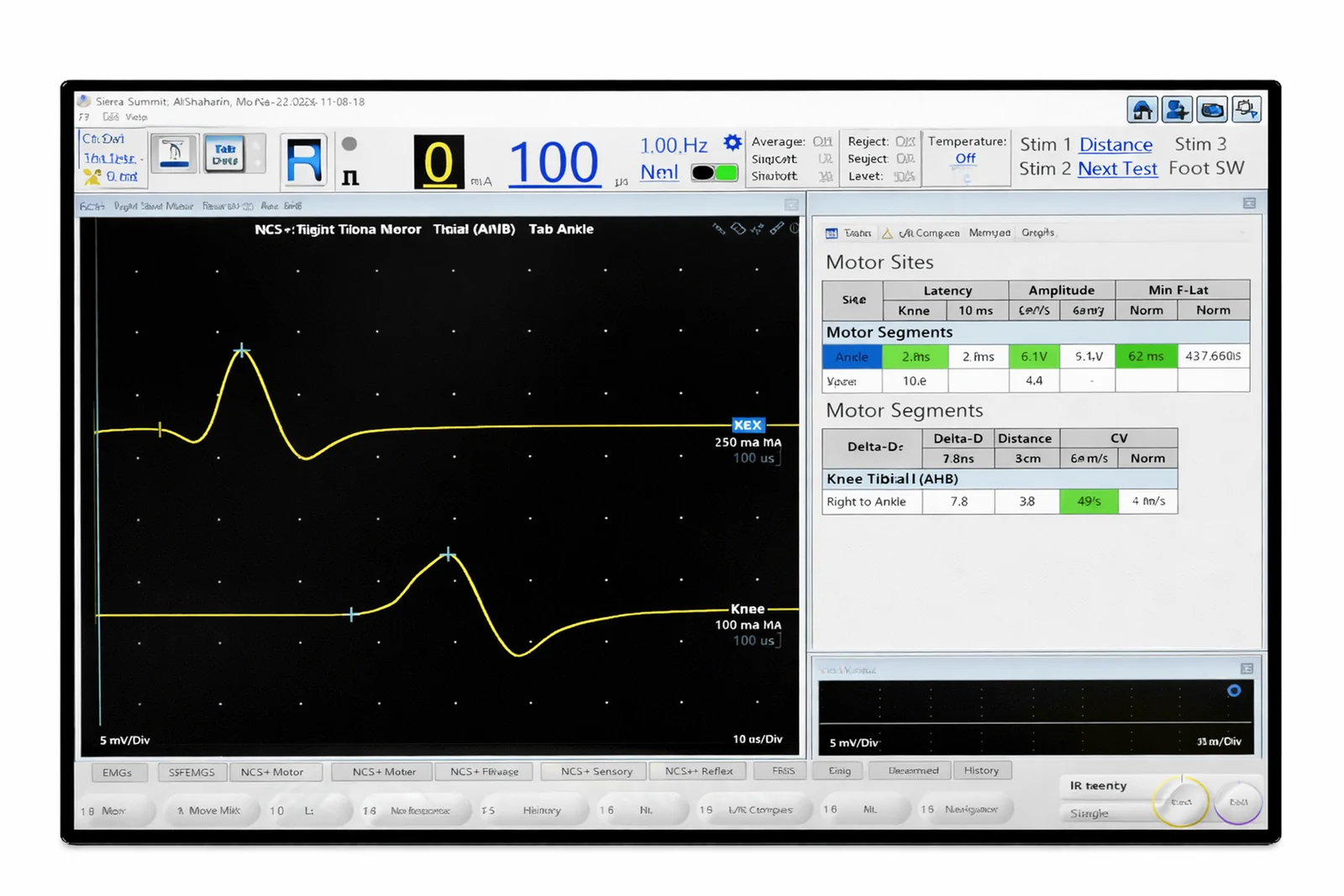
Right tibial motor nerve conduction study recording from abductor hallucis brevis (AHB) muscle: stimulation at ankle, and popliteal fossa

**Figure 7:**
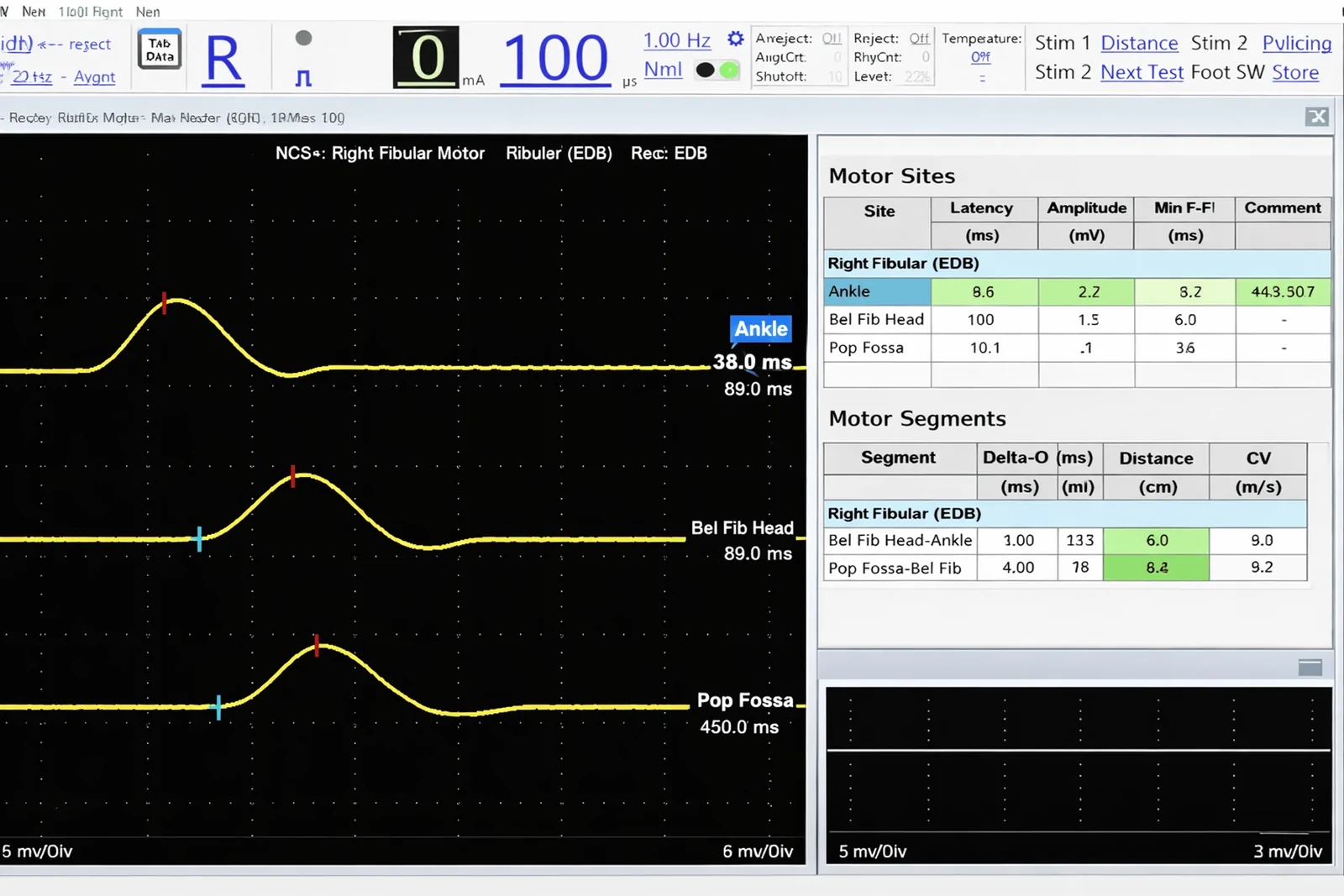
Right fibular motor nerve NCS recording from the EDB muscle. Stimulation sites: ankle, below fibular head, above fibular neck NCS: nerve conduction study; EDB: extensor digitorum brevis.

Figures 4-7 depict the results of the nerve conduction study of bilateral (left and right) motor tibial and fibular nerves. The study showed normal compound muscle action potential (CMAP) waves of both motor nerves at different stimulation sites with normal wave, latency, amplitude and conduction velocity (CV).

**Figure 8:**
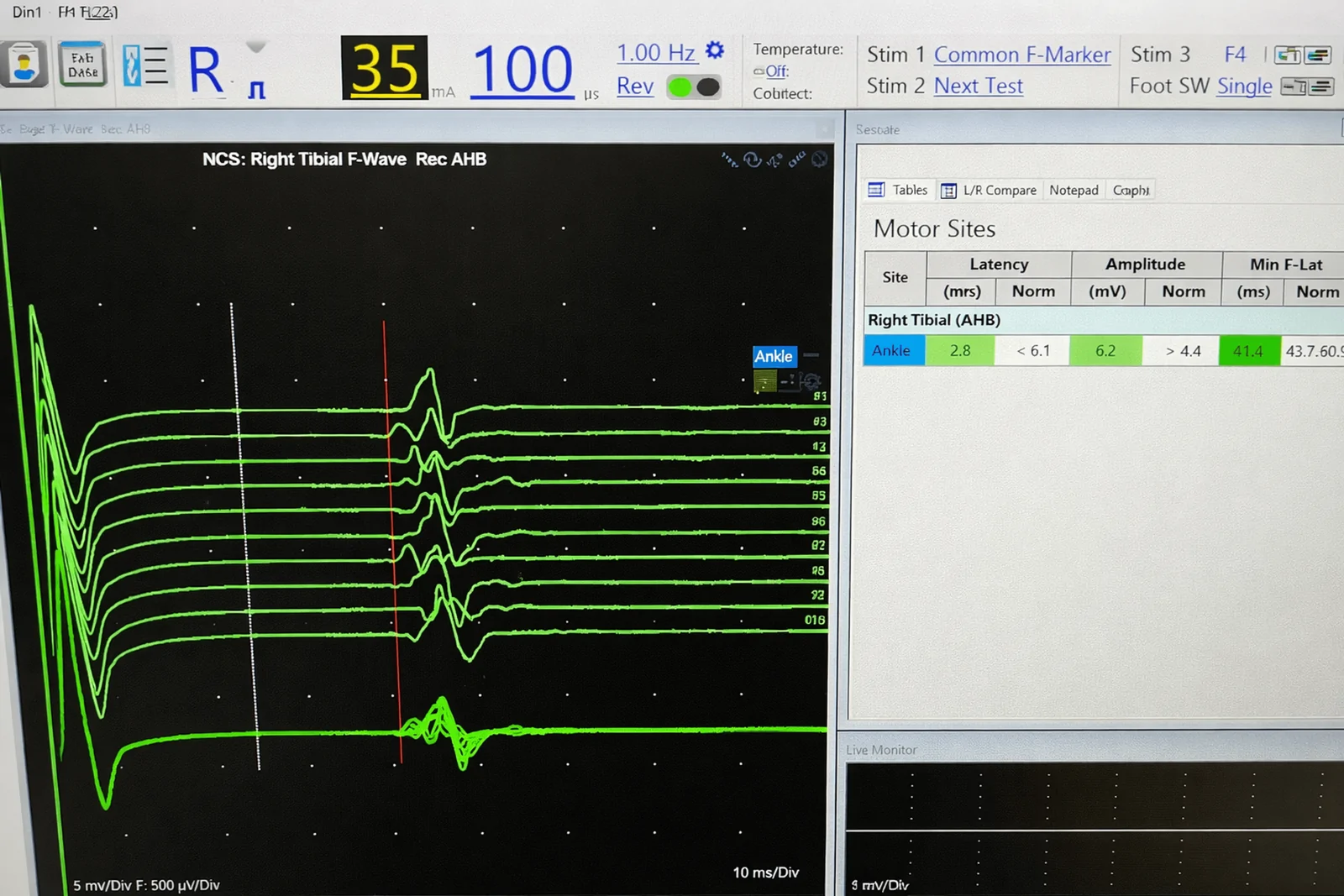
Right tibial F-wave. Recording from AHB muscle, stimulation at ankle AHB: Abductor hallucis brevis.

**Figure 9:**
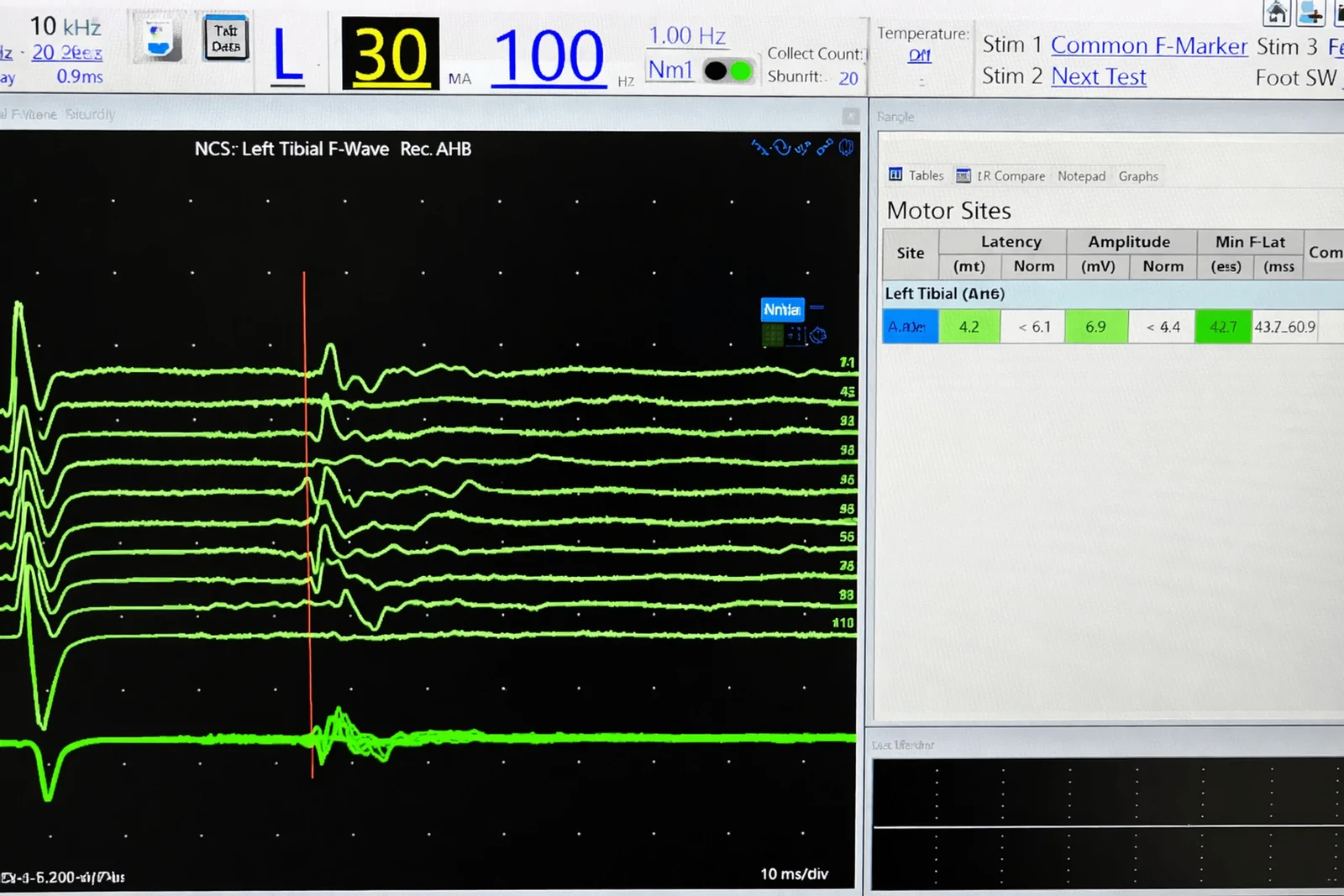
Left tibial F-wave. Recording from AHB muscle, stimulation site: ankle AHB: Abductor hallucis brevis.

**Figure 10:**
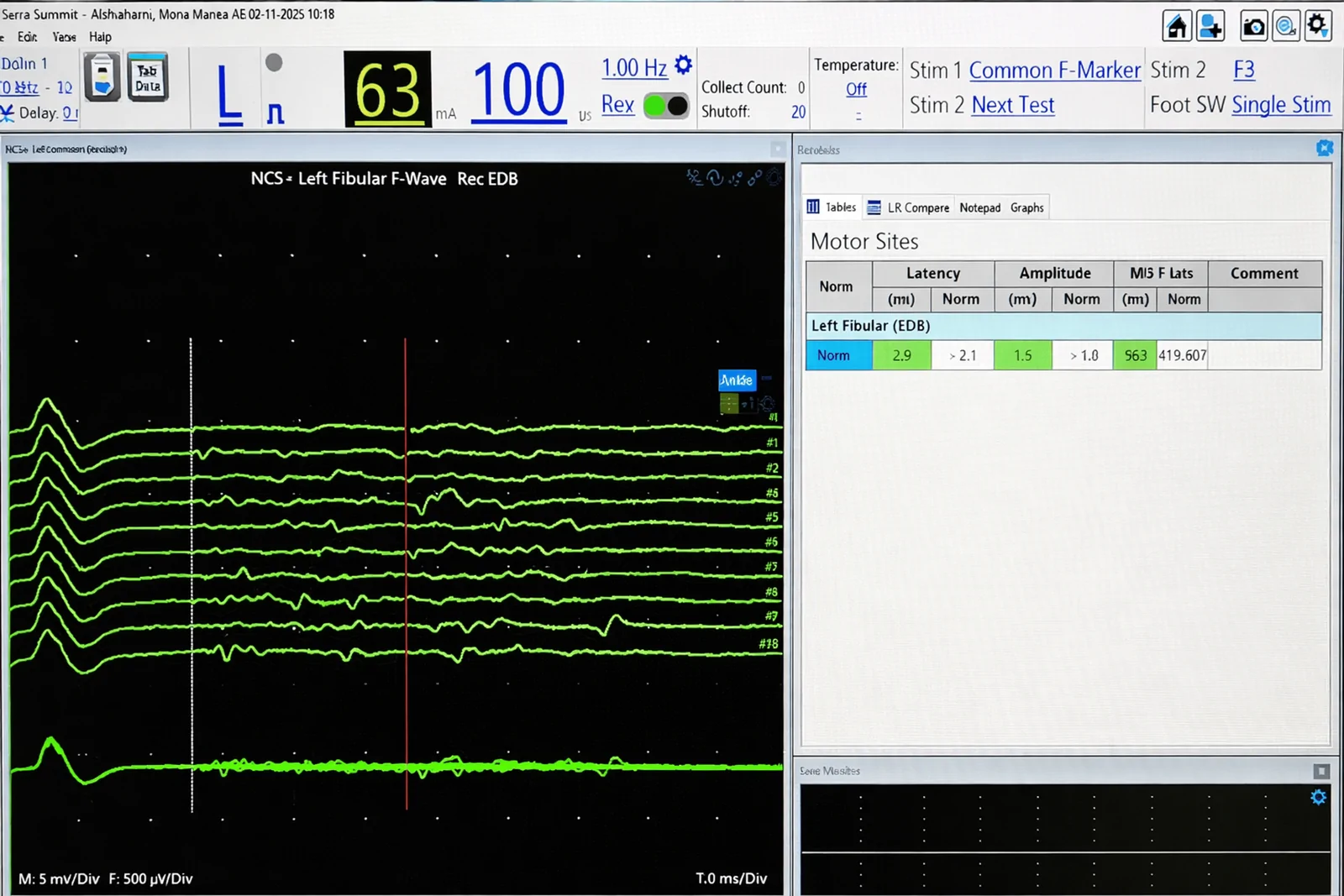
Left fibular F-waves recording from EDB muscle. Stimulation site: ankle.

**Figure 11:**
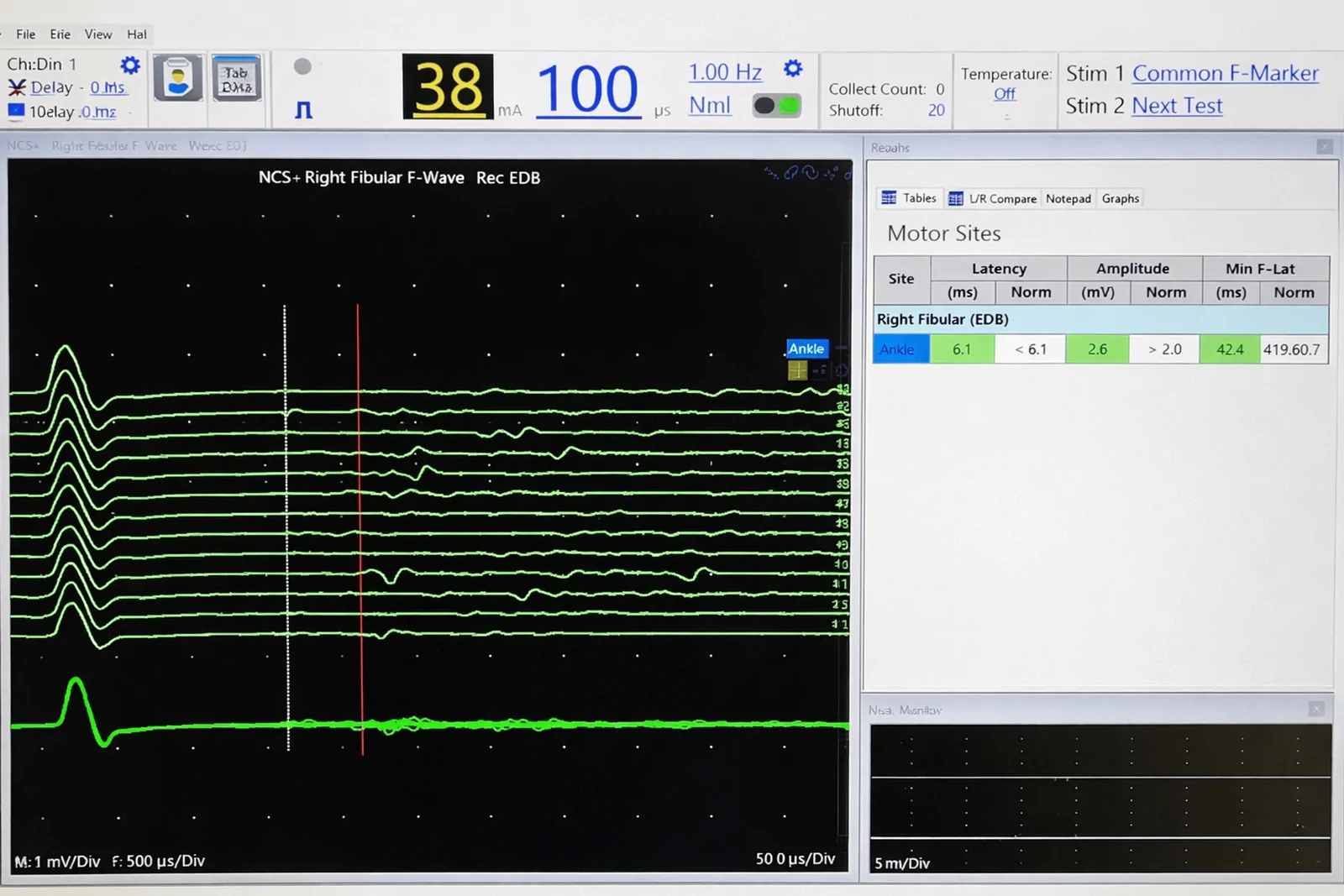
Right fibular F-wave. Recording from EDB muscle; stimulation site: ankle EDB: Extensor digitorum brevis.

Figures 8-11 show the F-wave recordings of bilateral tibial and peroneal nerves. The response was normal and normal latency values were observed, which indicate that there was no axonal damage of the proximal or distal nerves.

**Figure 12:**
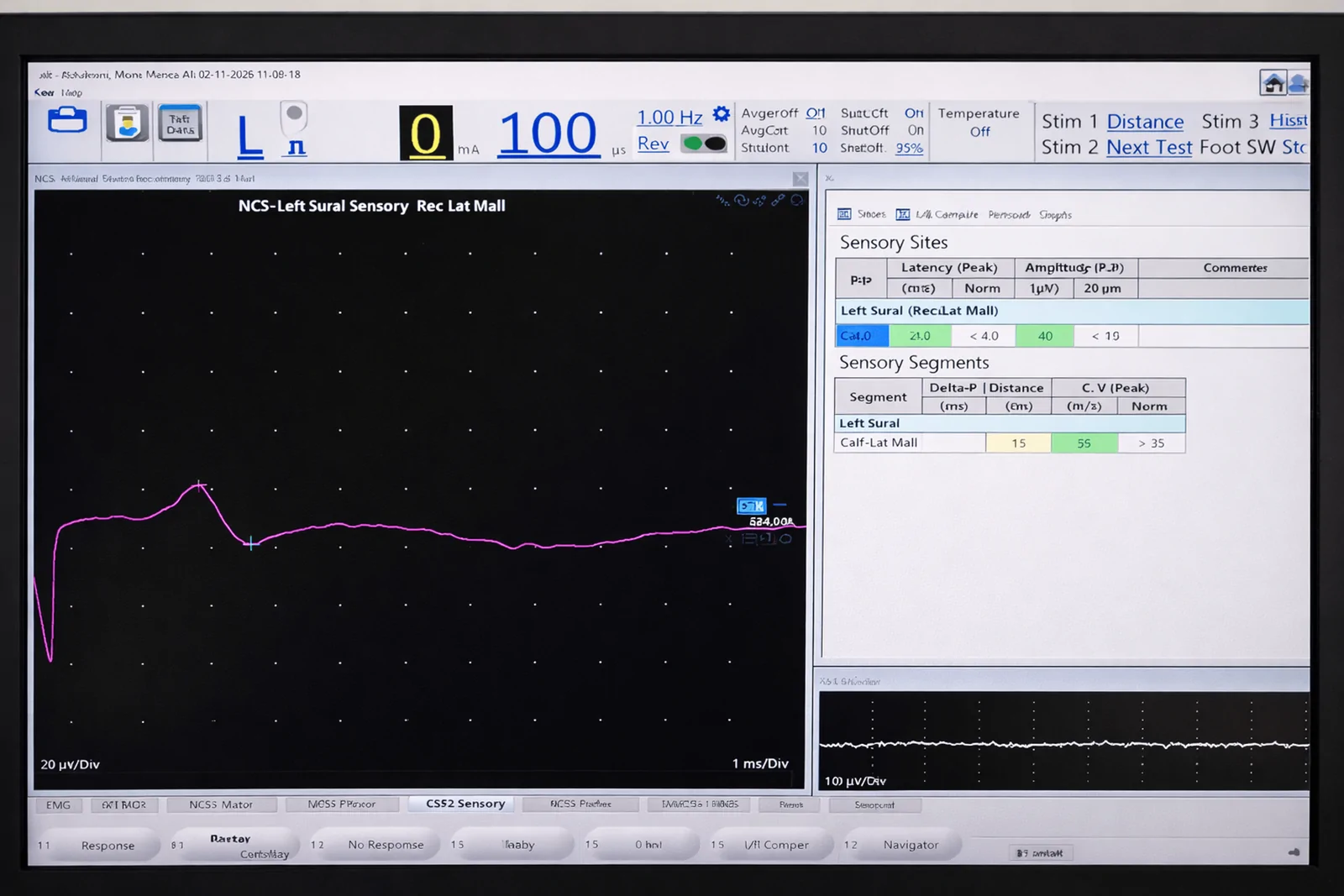
Left sural nerve (sensory) NCS. Recording site: posterior ankle; stimulation site: Posterior lateral calf. NCS: Nerve conduction study.

**Figure 13:**
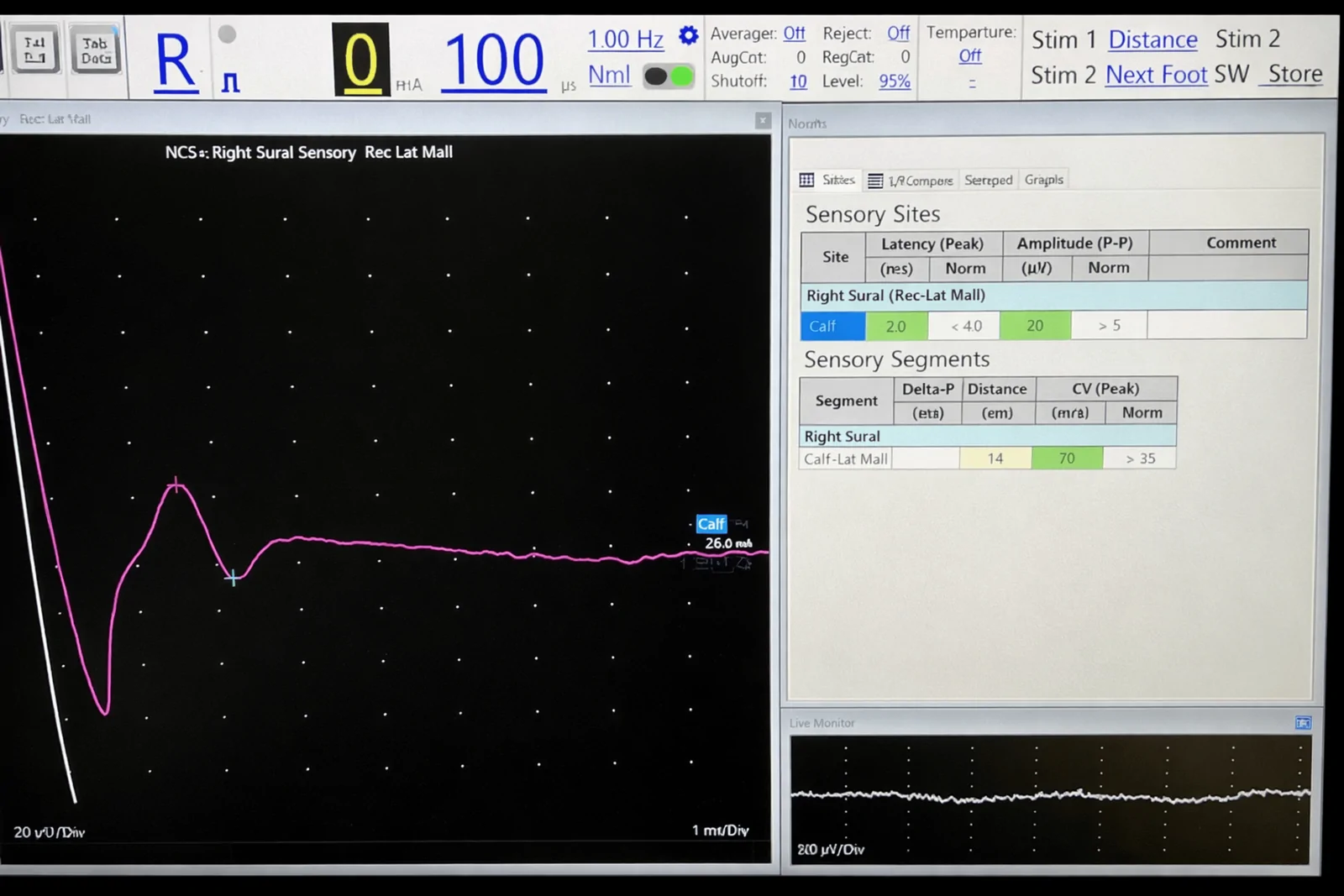
Right sural nerve (sensory) NCS. Recording site: posterior ankle; stimulation site: posterior lateral calf NCS: Nerve conduction study. This study indicates normal sural sensory nerve conduction study.

Figures 12, 13 represent the results of the right and left sural nerve conduction study, showing normal sensory nerve action potential wave (SNAP) with normal values of amplitude and conduction velocity.

Nerve conduction studies (NCS) of the lower limbs were performed to evaluate for neuropathy disorders. Motor and sensory nerve conduction parameters were within normal limits, with no evidence of demyelination or axonal loss. These findings ruled out the possibility of Friedrich ataxia, which may manifest as dysarthria, ataxia, and neuropathy [16].

Laboratory workup

Laboratory investigations are summarized in Table 3. All values were within normal limits and did not suggest a metabolic or systemic cause. Collectively, these findings were counterarguments to metabolic and systemic explanations of progressively dysarthric patients [17].

**Table 3:** Laboratory investigations WBC: White blood cells; TSH: thyroid-stimulating hormone; RBC: red blood cells; MCV: mean corpuscular volume; MCH: mean corpuscular hemoglobin; MCHC: mean corpuscular hemoglobin concentration; RDW: red cell distribution width; HDL: high-density lipoprotein; LDL: low-density lipoprotein.

Parameter	Patient Value	Reference Range
Glucose	5.8 mmol/L	3.4-8.7 mmol/L
Hemoglobin	152 g/L	135-180 g/L
WBC	4.9×10⁹/L	4-11×10⁹/L
Vitamin B12	253 pmol/L	138-652 pmol/L
TSH	3.58 mIU/L	0.35-4.94 mIU/L
Vitamin D	73.3 nmol/L	50-125 nmol/L
Alpha-fetoprotein	3 ng/mL	<10 ng/mL
Copper	13.43 µmol/L	12-18 µmol/L
Ceruloplasmin	0.28 g/L	0.20-0.60 g/L
RBC	5.1×10¹²/L	4.5-6.1 ×10¹²/L
Hematocrit (Hct)	0.453 L/L	0.42-0.54 L/L
MCV	89.1 fL	76-96 fL
MCH	29.9 pg	27-32 pg
MCHC	335 g/L	320-350 g/L
RDW	13.1%	11.5-14.5%
Total Cholesterol	4.45 mmol/L	<5.18 mmol/L
HDL Cholesterol	1.12 mmol/L	>1.55 mmol/L
LDL Cholesterol	2.98 mmol/L	<2.6 mmol/L
Triglycerides	1.81 mmol/L	<1.7 mmol/L
Uric Acid	375 μmol/L	210-420 μmol/L
Phosphorus	1.1 mmol/L	0.74-1.52 mmol/L
Adjusted Calcium	2.2 mmol/L	2.1-2.55 mmol/L
Vitamin E (α-tocopherol)	14.9 μmol/L	11.6-46.4 μmol/L
Ethanol	<2.1 mmol/L	<2.2 mmol/L
Toxicology screen (cannabinoids, cocaine, amphetamines, barbiturates, opiates, benzodiazepines)	Negative (all)	Negative

Genetic analysis

Since structural lesions were not detected on MRI, metabolic workup showed normal parameters. Given the strong family history of ataxia, genetic assessment was undertaken. Whole-exome sequencing (WES) was done. A homozygous identity nonsense mutation in the GDAP2 gene was recognized with bioinformatic analysis (c.475C>T, p.). Gln159*). This type of mutation introduces a premature stop codon in exon 5 of 14, which is expected to cause loss of functional protein and results in the production of a shortened protein that is likely non-functional.

Variant interpretation was performed according to the American College of Medical Genetics and Genomics (ACMG) guidelines [18]. The mutation's truncating nature is not present in population databases. The mutation had not appeared in the literature before, which means that it is a new addition to the mutational phenotype of SCAR27. The evidence that this mutation carries validity comes from the patient's brother, who has ataxia and slurred speech, was tested and showed a similar genetic mutation of GDAP2 gene c475C>T p.(GIn159*), which indicates that this variant is pathologic.

Differential exclusion

The results of imaging, laboratory, and electrophysiological studies together served to rule out a number of possible differential diagnoses. There were no signs of upper/lower motor neuron lesions on examination, which suggests amyotrophic lateral sclerosis. Multiple sclerosis, cerebrovascular disease, and structural cerebellar degeneration were eliminated by normal neuroimaging. The absence of metabolic abnormalities also ruled out vitamin E deficiency, Wilson disease, and any other treatable ataxia. The availability of an established pathogenic form of GDAP2 offered an established molecular diagnosis of an autosomal recessive spinocerebellar ataxia type 27 (SCAR27). The studies revealed that although MRI was normal and no specific laboratory results were detected, genetic tests played a pivotal role in establishing the diagnosis. Given these conditions, further genetic investigation in patients with unexplained progressive neurological symptoms is especially noteworthy when a positive family history is present.

## Discussion

SCAs are genetically heterogeneous group of neurodegenerative diseases, and there are over 40 distinct subtypes of the disease. They are usually characterized by progressive gait disturbance, limb incoordination, and cerebellar dysarthria with a wide variability in the constellation of symptoms according to the underlying gene defect. Autosomal recessive spinocerebellar ataxias (SCARs), which are lower than the autosomal dominant forms, are increasingly recognized with the advent of next-generation sequencing technologies. Among them, spinocerebellar ataxia type 27 (SCAR27) is linked to a pair of mutations (doublets) in the gene GDAP2, which was relatively recently described and which is a bit of a gene that just started to make physiological sense [6,19].

Patients with GDAP2-associated ataxia typically start experiencing symptoms in adolescence or early adulthood and include symptoms such as instability with walking, dysarthria, and other signs seen in cerebellar dysfunction. In several cases, clinical worsening is accompanied by spasticity, cognitive impairment, and structural cerebellar atrophy on MRI. However, there is an important phenotypic variability. The case here is interesting in that it shows the predominance of progressive dysarthria over several years, with subtler cerebellar ataxia and normal neuroimaging during earlier stages of the disease.

Such a presentation is not the usual course of the hereditary ataxias. Often, the gait disturbance is the earliest and most disability-causing feature. The predominance of GDAP2 mutations points to the heterogeneous nature of the expression of the GDAP2 mutation and indicates a predisposition of definite cerebellar circuits to disease in certain genetic backgrounds, especially those connected with motor speech coordination.

Comparison with the reported literature

Since it was first described, the phenotypic spectrum of GDAP2-associated spinocerebellar ataxia has been reported in very few instances. Breza et al. (2020) studied homozygous GDAP2 mutations, which were found to cause juvenile-onset cerebellar ataxia, characterized by prominent gait instability and dysarthria, and the disease mechanism was determined to be a loss of function [20]. They made multiple points about variable presentations that manifest between adolescent and adult onset, which were often characterized by cerebellar atrophy on MRI and, in some cases, cognitive impairment [21]. GDAP2-related ataxia has been reported with variable clinical presentations, including adult-onset cerebellar ataxia [20].

The common idea in these reports is that gait ataxia and limb incoordination are usually the first and most debilitating manifestations, and dysarthria, albeit frequently, is often comorbid but not overriding. Conversely, the current case has shown a phenotype of dysarthria being more prominent and dominant over a span of several years before gait disturbance, which highlights the heterogeneity of the GDAP2-related disease expression. The outcomes of neuroimaging are also dissimilar. In the majority of the reported cases, cerebellar atrophies were severe. Still, MRI was confirmed as normal in this patient, even after years of symptom experience had passed, which indicated any structural change might be delayed by impaired relative functionalization, and even absent in some [21]. Furthermore, it did not have the extra-cerebellar manifestation (i.e., spasticity or cognitive impairment) as described in other cohorts and was not evident in this instance, which suggests a milder clinical outcome.

The frequency of all these comparisons demonstrates that GDAP2 mutations can lie in the range of the extreme clinical phenotypes, including intractable early-onset ataxia and neuroimaging abnormalities, as well as milder phenotypes with isolated dysarthria. This underlines the need to document the atypical cases so as to concentrate on the genotype-phenotype relationship and also to identify SCAR27 in other clinical locations.

Diagnostic challenges

Middle-aged progressive dysarthria is typically attributed to more common conditions such as cerebrovascular disease, motor neuron disease, or demyelinating disorders. The first impression, once created by routine imaging and neurophysiological tests in the given case, was indicative of a hereditary ataxia.

Usually, the family members carry similar findings and course progression, which is not found in this case. Despite such indications, genetic testing should also be an earlier diagnostic tool for clinicians, particularly in the case of people who develop neurological disease with a previous history in their family [22].

Genotype-phenotype correlation

The range of the clinical manifestations of GDAP2 mutations leads to significant concerns about how the phenotypes and the genes are related [23]. Patients have shown evident variations in the age of onset and in the distribution of clinical manifestations, even though the protein activity of GDAP2 is always compromised [24]. In cases where mutations are confirmed as loss-of-function, such mutations consistently initiate the disease process and drive its progression. In some individuals, cerebellar atrophy appears on MRI, with juvenile-onset cerebellar ataxia and a fast course, and in other individuals in middle adulthood with partially isolated dysarthria or gradually progressive gait instability [25]. This broad range implies that other genetic, epigenetic, or environmental factors contribute to the development of the illness, rather than being determined by the presence of the mutation [2].

The modification of mitochondrial activity, oxidative stress response, or synaptic communication can elevate or mitigate the role of GDAP2 malfunction using modifier genes. Although environmental exposures (such as lifestyle, pollutants, or co-morbid medical conditions) may influence the course of disease progression, epigenetic factors, including DNA methylation and histone modification, may further determine neuronal susceptibility [26]. However, the current situation emphasizes the need to carefully record new variants and related phenotypes to improve the associations between the phenotype and the genotype. Such information may help doctors to forecast the most likely clinical outcome and improve the predictive power of genetic counseling. Moreover, a new set of presentations to the collection offers meaningful new information on selective cerebellar vulnerability and helps to guide customized treatment provisions. Therefore, the sensitivity of the diversity of SCAR27 is not only important in diagnosing it appropriately, but also in the direction of future research and family therapy [10].

Clinical implications

There are several practical implications associated with the identification of a new GDAP2 mutation. First, it extends the phenotypic range of SCAR27 to include phenotypes where progressive dysarthria may be the onset of gait ataxia. This is why clinicians should never overlook the possibility of hereditary ataxia among patients with progressive speech disorders. Second, the exclusion criterion is not restricted to the imaging abnormalities, in terms of considering a genetic diagnosis. Third, the case highlights the importance of family history: the discovery of an affected sibling was also important to begin the diagnostic process.

Genetic confirmation also has implications for patient care, and the same applies to family counseling. Even though there is no curative prophylaxis, due to appropriate diagnosis, it provides psychological closure, prognosis, and helps one decide concerning reproduction. Moreover, patients with a genetic diagnosis will be able to join clinical trials or such research opportunities that explore the specific background for ataxia therapies.

Broader research and therapeutic perspectives

In research terms, further studies where GDAP2 variants are reported help maintain a more extensive picture of the role of the gene in cerebellar functioning and neurodegeneration. The role of GDAP2 in adaptation to stress and mitochondrial homeostasis is rather clear; however, the specific mechanism of stress adaptation remains unclear. Additional functional investigations would also be required to interpret the causes of the listed mutations to produce selective weakness of cerebellar neurons.

Clinically, an understanding of the involvement of stress-response mechanisms in GDAP2-related ataxia creates the possibility of an intervention to improve neuronal tolerance to oxidative stress. Although the present management approach is favorable, potential future therapeutic approaches involve gene replacement, RNA-based therapies to overcome nonsense mutations, or pharmacological compounds to enhance mitochondrial performance and stress adaptation mechanisms.

Limitations of the case

Like all case reports, there are some limitations. Genetic testing was confined to the proband, and there was no segregation testing of other family members. There were no functional studies that directly tested the nature of the effect of the identified mutation on protein expression. However, the clinical picture, positive genetic mutation in two symptomatic siblings, and classification of the variant as potentially pathogenic are strong reasons to assert its role in the occurrence of the disease.

## Conclusions

This case describes an atypical presentation of SCAR27 associated with a novel nonsense mutation in the GDAP2 gene. Unlike most reported cases where gait ataxia and cerebellar atrophy dominate the clinical picture, this patient exhibited progressive dysarthria as the earliest and most disabling symptom. In contrast, gait disturbance remained mild, and neuroimaging showed no significant abnormalities. Such a presentation expands the phenotypic spectrum of GDAP2-related disorders and highlights the variability in disease expression. The identification of the c.475C>T (p. Gln159*) mutation, previously unreported in the literature, reinforces the role of next-generation sequencing in uncovering rare hereditary ataxias when conventional investigations are inconclusive. Genetic confirmation not only provides diagnostic certainty but also guides counseling regarding prognosis, inheritance, and recurrence risks. This case emphasizes the importance of considering hereditary ataxias in the differential diagnosis of unexplained progressive dysarthria. It demonstrates how careful clinical evaluation combined with genomic testing can advance diagnostic accuracy, inform patient care, and contribute to broader research efforts.
